# Inkjet printing of plasma surface–modified wool and cotton fabrics with plant-based inks

**DOI:** 10.1007/s11356-022-20659-3

**Published:** 2022-05-10

**Authors:** Alka Madhukar Thakker, Danmei Sun, David Bucknall

**Affiliations:** 1 School of Textiles and Design, Heriot-Watt University, Edinburgh, UK; 2 School of Engineering and Physical Sciences, Heriot-Watt University, Scottish Borders, UK

**Keywords:** Plasma surface modification, Wool, Cotton, Inkjet printing, Herbal inks, Herbal fabrics, Sustainable textiles

## Abstract

In this research paper, sustainable technologies that are plasma surface treatment and digital printing of wool and cotton fabrics with herbal inks are implemented for ecological outcomes. One of the significant objectives was to study the plasma surface modification and its implication on fabric absorbency, K/S values gained, and the fastness properties of the resultant herbal fabrics. The wash fastness to colour change was studied wherein plasma surface treatment remarkably improved wash fastness ratings from 1–2 to 3–4 obtained on inkjet printed wool and cotton fabrics. These findings were supported by data gained from optical tensiometer, ATR-FTIR, drop test and SEM justifying the enhanced wettability of the modified fabrics. The factorial experiment was designed for this segment of research, and it was further validated with ANOVA one-way test. The concluding parametric study with plasma surface modification yielded the probability value of 0.000463 and actual power of 0.99 which is reassuring. The ecological characterisation and assessment of functional properties of the herbal fabrics are suggested for the forthcoming study.

## Introduction

Conventional surface modification techniques of textile substrates with petroleum-based raw materials are high in global warming potential value (Palaskar and Desai 2016). Therefore, to combat the problem, the mechanical surface modification method ought to be encouraged such as plasma surface modification technology. The surface morphology and the structure of the wool fibre are complex. Chemically, about 80% of wool is keratin proteins, which are condensed amino acids (Mather and Wardman 2011). The structure of wool fibre consists of an outer layer cuticle (10%) and the inner cortex (85%). The scaly cuticle consists of an epicuticle, exocuticle and endocuticle. The epicuticle is predominantly hydrophobic keratin and lipids; it is a matter of concern as it affects absorption during subsequent processes of dyeing and printing. The traditional process of removing epicuticle is by chlorination which is unsustainable. Eco-friendly plasma treatment is widely experimented with to introduce hydrophilic polar groups such as − OH, − C = O, and − COOH onto the fibre surface. Plasma treatment is also noted to attack disulfide crosslinks oxidizing to sulfo-sulfonate and cysteic acid moieties (Mather and Wardman 2011). The research was performed with oxygen plasma treatment on wool yarns. The 5 min of oxygen plasma treatment was found to have enhanced yarn surface roughness. The images made by a high-resolution scandisk confocal microscopy revealed an increased ingrained structure of wool fibres after 5 min of oxygen plasma treatment as compared to the untreated. The enhanced grooves due to shrinkage increased the absorption capacity of wool fibres (Barani and Calvimontes 2014). However, the study did not justify the enhanced hydrophilicity by performing further wet processing namely dyeing or printing or wettability tests such as surface contact angle measurement and drop-test. To further reinforce the plasma surface modification of wool fabric surface, Singh et al. performed pre-mordanting of wool fabric with biomordant tannin extracted from the tamarind seed coat and naturally coloured with kapok flower extract. The treated wool fabric exhibited excellent K/S values, fastness properties, anti-bacterial and antioxidant functionality (Singh et al. 2019). Data from several sources have identified the increased CO–, COO– and –OH polar groups on wool fabric substrate credited to plasma surface modification technology (Sun and Stylios 2006).

Similarly, several studies have postulated a convergence between plasma surface modification of cotton and increased wettability for an improved colour yield of the treated fabric. Molina et al. performed an experiment on cotton fabric pre-treated with water, deuterated water and ethanol, and subsequently treated with helium plasma gas. The X-ray photoelectron spectroscopy (XPS) and Fourier transform infrared spectroscopy (FTIR) identified the presence of additional functional groups of C–O, C = O and O–C = O, hence improving the surface properties of the cotton fabric effectively. The study inferred that cotton fabric soaked with deuterated water and sequentially treated with helium plasma-treated oxidized the cotton fabric efficiently, as compared to dry helium plasma treatment. However, both helium and deuterated water are expensive; therefore, the study would have limited applications (Molina et al. 2020). Considering free and sustainable “air” plasma surface treatment in the study would be a holistic approach as it inherently contains oxygen, nitrogen and water vapour. On the other hand, Pransilp et al. performed plasma treatment on cotton fabrics with oxygen, nitrogen and sulfur hexafluoride gases; the treated fabrics were further analysed by XPS. The cotton fabric treated with oxygen plasma gas exhibited less proportion of C–C and C–H groups and an increased ratio of C–O and C–OH functional groups than the untreated. In addition, O–C = O functional group was noted in the treated fabric. The treated fabric had greater wettability in comparison to the untreated. These polar groups also act as an anchoring site for other functional groups (Mather and Wardman 2011) (Chi-wai 2015). A type of oxygen gas plasma reaction occurred wherein oxygen gas molecules, O_2_ ionized to O^+^ and O^−^ on collision with cotton cellulosic polymer. The C–C bonds in the cotton fibres ionize due to which O = C–O bonds are formed. These results are parallel to those reported by Sun and Stylios (Sun and Stylios 2006) (Porntapin et al. 2016) (Chi-Wai and Wai-Shan 2018). This explains how oxygen plasma–treated cotton fabric surface acquires greater hydrophilicity and K/S values (Porntapin et al. 2016). Vaideki et al. concluded that cotton fabric treated with radiofrequency oxygen gas plasma for 10 min had higher hydrophilicity, so enhanced absorption of herbal neem extract solution (Vaideki et al. 2008). On the contrary, Koh et al. analysed argon gas plasma surface-modified cotton fabric–denoted greater wettability and absorption of gallnut extract solution than oxygen gas plasma surface-treated fabric (Koh and Hong 2014). However, it is to be noted that argon is a noble gas and hence expensive.

Additionally, the life cycle analysis report of plasma-treated PET fabric on the environment showed a remarkable percentage saving of energy, water, COD, BOD, CO_2_ and CO as compared to conventional chemical processing (Palaskar and Desai 2016). Plasma treatment profoundly lowers energy, time and chemical consumption so reduced effluent load and decreased the Global Warming Potential (GWP) value (Palaskar and Desai 2016) (Chi-wai 2015). Together, these studies provide important insights into the role of plasma surface modification on cotton fabric and conclude air plasma surface modification to be one of the most viable and sustainable treatments to improve the surface energy of a textile substrate. The plasma surface treated fabrics could be examined for modifications in surface morphology of wool and cotton fabrics with the Attenuated Total Reflectance-Fourier Transform Infrared Spectroscopy (ATR-FTIR), Drop-test, scanning electron microscope (SEM) and surface contact angle.

**Fig. 1:**
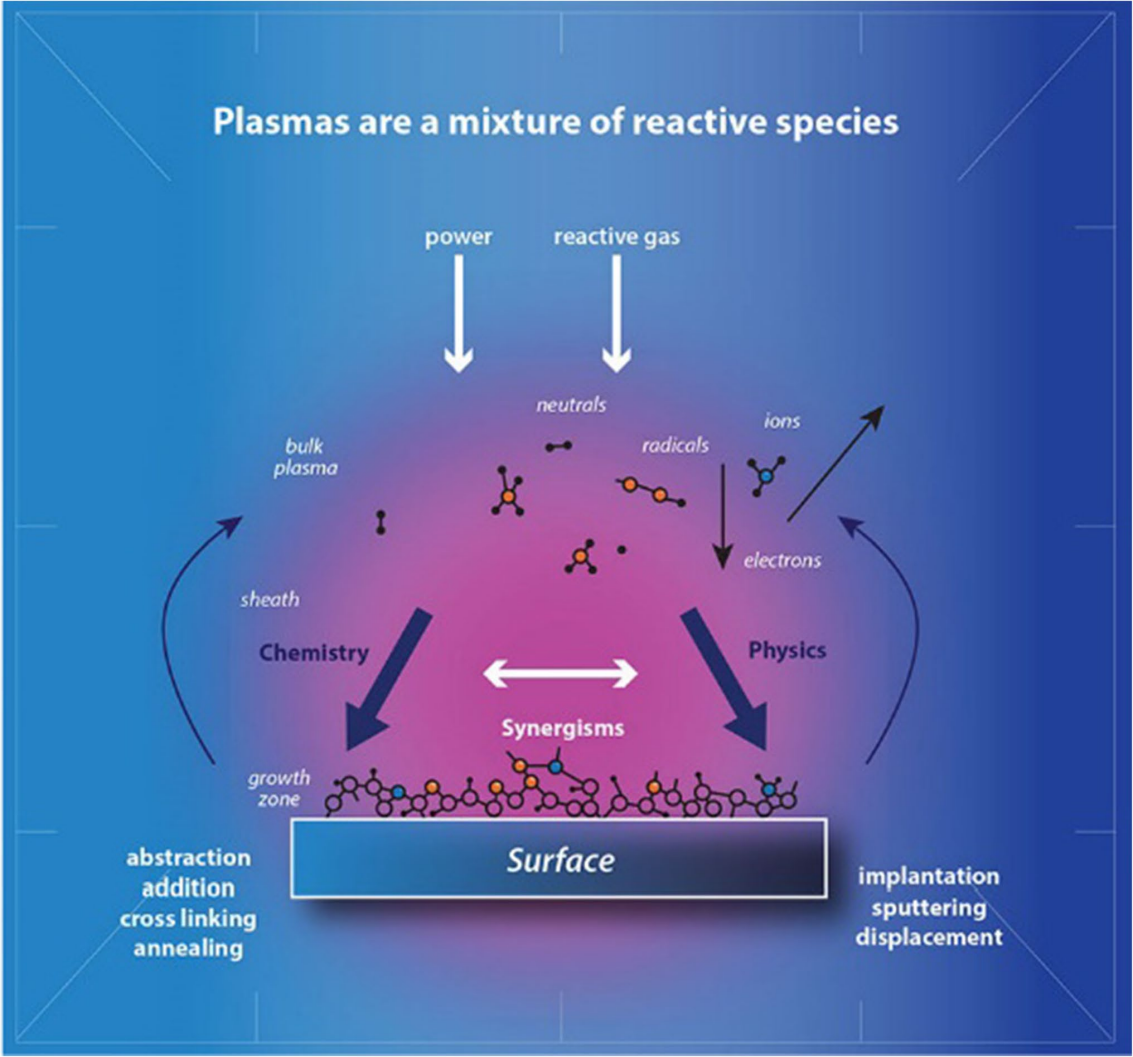
Air plasma surface activation of the substrate surface (Henniker Plasma 2021)

Typically, plasma is known as the fourth state of matter (by energy); it is everywhere in the universe (Palaskar and Desai 2016). The plasma atmosphere consists of free electrons, radicals, ions, atoms, molecules and several different exciting particles independent of the employed gas. The plasma surface treatment of the substrate increases the surface energy of the substrate by activating its atoms and molecules, as demonstrated in Fig. 1. This is accomplished by bombarding the substrate with free radicals/ions/electrons from plasma gases (such as oxygen, nitrogen, others), resulting in new functional groups conferring new properties to the treated substrates (Mather and Wardman 2011) (Chi-wai 2015) (Plasma Etch 2020) (Henniker Plasma 2021) (Barani and Calvimontes 2014). The video in (Henniker Plasma 2021) efficiently demonstrates the process of plasma surface modification (PSM). The benefit of plasma treatment is that the surface molecular properties can be changed significantly over a short period of treatment. Vacuum packing the treated material would prolong the shelf life of plasma (Plasma Etch 2020). It is anticipated that in this manner, the plant-based inks would adhere to the PSM wool and cotton fabrics. In summary, the phytochemicals polar groups in plant-based inks constituting chromophores and auxochromes play a vital role in covalent bond formation, with the fibre structure (with enhanced polarity and anchoring sites due to PSM) eventually responsible for the colour values obtained, fastness properties and functional properties of the resultant sustainable fabrics. The improved bond formation occurs due to PSM. The designed research implements sustainable technologies, materials and methods. The sustainable inputs assure sustainable outputs that would benefit the environment and human health alike.

## Materials and methods

### Materials

The research adheres to sustainable fabrics that are wool and cotton as specified in Table 1. Also, the commercially pre-treated wool and cotton fabric samples were utilised in the study. The plasma surface–treated fabrics and commercial pre-treated fabrics were digitally printed with herbal inks for comparative analysis. All fabrics for the study were sourced from Whaley’s Bradford Limited, UK.

**Table 1:** Wool and cotton fabric specifications

Fabric	Weave structure	Fibre type	Yarn count, tex		Density		Fabric weight, g/m^2^
			Warp	Weft	Warp, ends/5 cm	Weft, picks/5 cm	
Wool	Plain weave	100%	26	26	65	50	115
Calico	Plain weave	100%	30	30	60	60	140

### Fabric specification

The herbal inks were formulated from plant extracts of bio indigo, quebracho red and the flame of the forest herb for blue, red and yellow colours correspondingly. The black herbal ink was constituted from a mixture of primary colours that are bio indigo, quebracho red and the flame of the forest herb. The rheological properties of the herbal inks are organised in Table 2. It was applied in research for the inkjet printing of plasma-treated and untreated wool and cotton fabrics.

**Table 2:** Physical properties of herbal ink

Herbal ink colour	Relative density	Viscosity (cP)	Surface tension (mN/m)	Conductivity (mS/cm)	pH
Distilled water	1	4.07	74	00.0	6.34
Bio indigo, C	1.06	7.67	58	09.6	7.69
Quebracho red, M	1.06	8.16	54	02.5	5.21
Sacred tree, Y	1.06	9.66	49	04.2	5.47
Bio indigo + quebracho red + sacred tree, K	1.06	9.46	59	10.3	5.88

Colours: C is cyan colour, M is Magenta, Y is yellow, and K is black

### Herbal inks

**Fig. 2:**
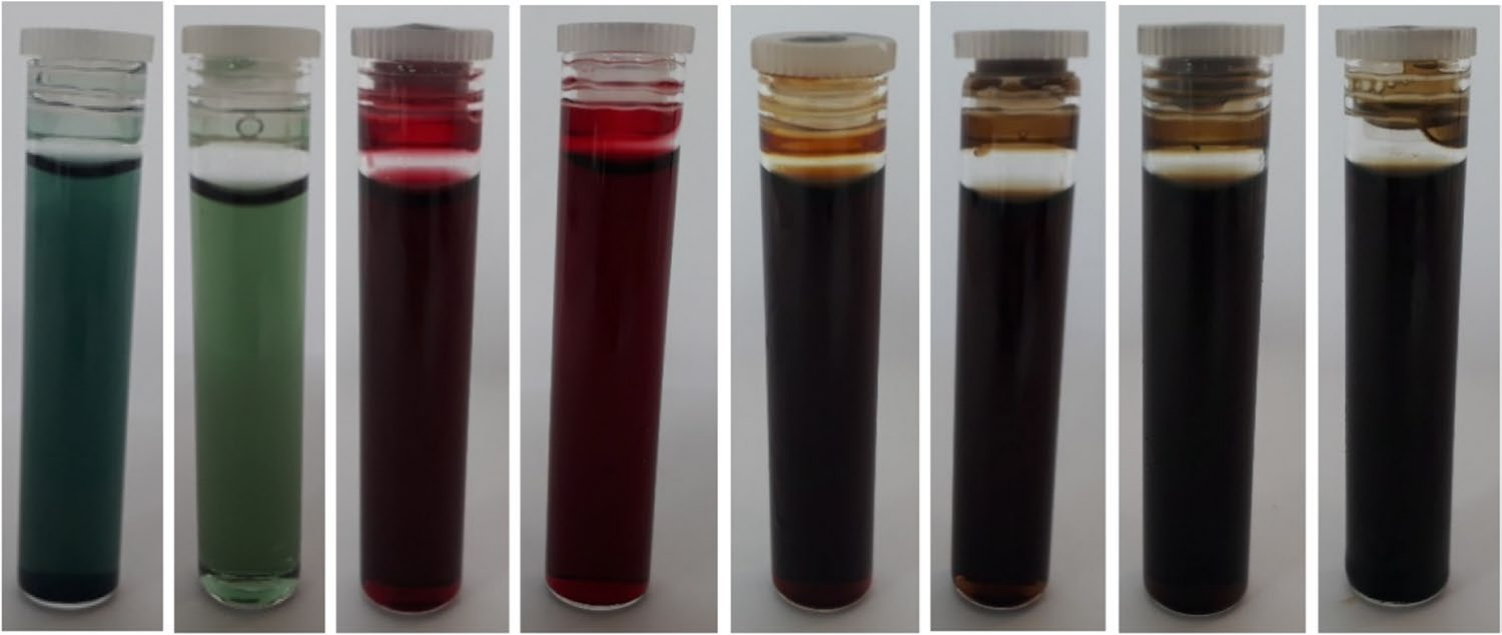
Double-filtered plant-based inks from left to right: cyan colour, light cyan colour, magenta colour, light magenta colour, yellow colour, black colour, light black colour and light, light black colour inks

The plant extract, distilled water and glycerol were taken by weight when preparing plant-based ink. The procured plant extracts were commercially extracted on the Soxhlet apparatus and ball-milled, typically utilising distilled water as solvent. The plant extracts were water-soluble; still, the milling was performed with hand pestle mortar. It was done to facilitate the dispersion of plant extract powder into distilled water. The milled plant extract was gradually added to the distilled water and simultaneously stirred on a magnetic stirrer at elevated speed for 2 min. The stirred mixture was allowed to stand still for 1 h for the surplus plant extract particles to settle down. The mixture was strained and filtered using a 70-mm Whatman glass fibre filter to obtain a pure colour solution. The filter was changed in between to eliminate the sludge obtained. The weigh up amount of glycerol was stirred into the filtered colour solution on a magnetic stirrer for 2 min at an elevated speed to obtain a homogenized solution. The resulting solution was filtered again through a microfilter (MF) — Millipore, 0.22 µm membrane (MCE) microfilter to ensure thorough filtration and homogenization of the colloidal solutions of plant-based inks. The sterile microfilters were 47-mm hydrophilic nano filters. Thereafter, the double-filtered ink was transferred into a sterile, air-tight bottle and labelled appropriately as illustrated in Fig. 2. The entire process was repeated to obtain eight inks based on the C, M, Y and K colour model of the printer. When working with small quantities of inks for percolation, the larger type of vacuum filtrations method was overlooked to prevent the potential risk of ink wastage. Also, the sludge formation during purification would inhibit the implementation of vacuum filtrations. Each of the synthesized ink was further examined for various rheological properties. The sludge, aka marc (Blankespoor n.d), is the leftover moistened plant materials that go idle due to achieved saturation and larger particle size of the plant extracts that could not pass through the filter. It is essential to eliminate the sludge and double filter the colloidal plant-based ink solution to prevent inkjet blockage. In other words, sludge is an organic by-product of the filtration process (Dictionary.com, LLC 2010); it is recyclable and biodegradable. The rheological properties of the constituted plant-based inks are summarised in Table 2.

## Methods

### Plasma surface modification of wool and cotton fabrics

**Fig. 3:**
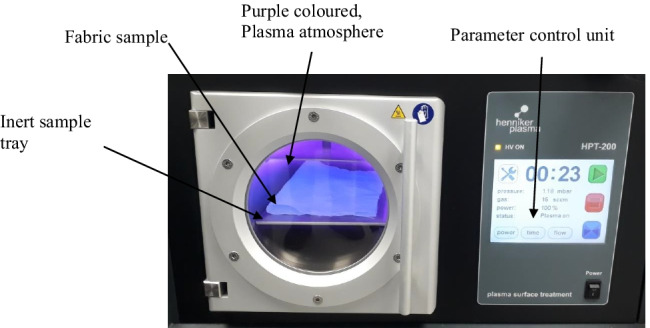
Henniker plasma treatment device illustrating surface activation of fabric

The plasma surface modification of wool and cotton fabrics was performed in vacuum plasma cleaner HPT-200 as demonstrated in Fig. 3. The process was performed at the Laboratory of Polymer Science, Heriot-Watt University, Edinburgh Campus.

**Fig. 4:**
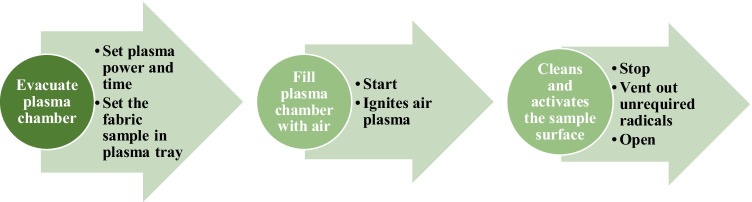
The steps followed in the plasma surface modification technique

The steps involved in plasma surface modification (PSM) of wool and cotton fabrics are depicted in Fig. 4.

Firstly, fabric samples were placed on the inert tray within the plasma surface treatment chamber. The plasma surface treatment (PST) parameters include gas type, gas flow (in sccm, is the standard cubic centimetre per minute (cm^3^/min) of gas flow), pressure, power level and treatment time that are shown in Table 3. Then, the mechanism is set ON and the operation begins by evacuating the plasma chamber of foreign particles and subsequently filling up with the compressed air from the external atmosphere for performing the PST. During the PST, the fabric surface is cleaned and activated. At the set time, the procedure ends and vents out the surplus radicals and indicates to open the device door. The plasma surface treated and untreated wool and cotton fabrics were further examined on an ATR-FTIR, optical tensiometer and SEM to examine chemical and physical changes of the treated fabric surface.

**Table 3:** Plasma treatment parameters

Air PST specifications	Value
Gas type	Atmospheric air
Gas flow, sccm	15
Pressure, mbar	1.67–2.26
Time, minutes	2, 3
Power, Watt	50, 100

### Fabric wettability test with tensiometer

**Fig. 5:**
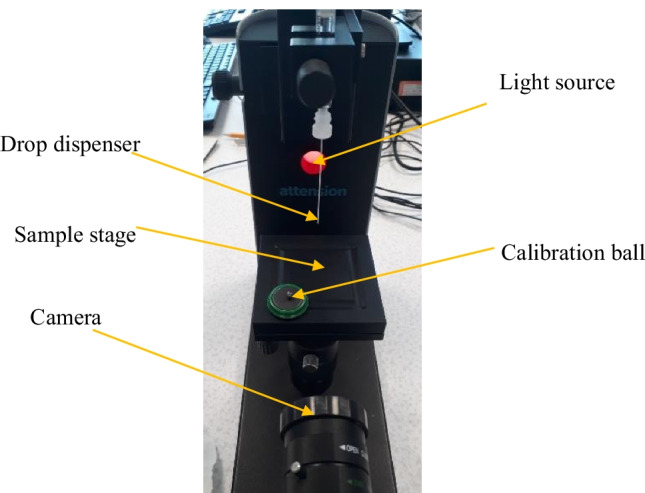
One attension optical tensiometer for surface contact angle measurement

An optical tensiometer also called a contact angle goniometer was utilized for calculating the surface contact angle (wettability) of the plasma-treated wool and cotton fabrics in comparison to the untreated. It consists of a camera, a dispenser to dispense a drop, a sample stage, and a light source to illuminate the drop on the sample stage as shown in Fig. 5. The optical tensiometer was calibrated with ball calibration to 143.60° baseline. Sessile drop measurement was utilized for surface contact angle measurement. In practice, the distilled water was dropped on the tested sample surface, and an image of the drop was recorded. The static angle is then defined by fitting the Young–Laplace Eq. (1) around the droplet:

1 $${\sigma }_{sg}={\sigma }_{sl}+{\sigma }_{lg}\times cos\theta$$

where, *θ* is the surface contact angle, *σ*_*sg*_ is the surface free energy (SFE) of the solid material, *σ*_*sl*_ is the interfacial tension of the solid material and the liquid, and *σ*_*lg*_ is the surface tension of the liquid (Kruss n.d).

### Surface morphology examination with scanning electron microscope

**Fig. 6:**
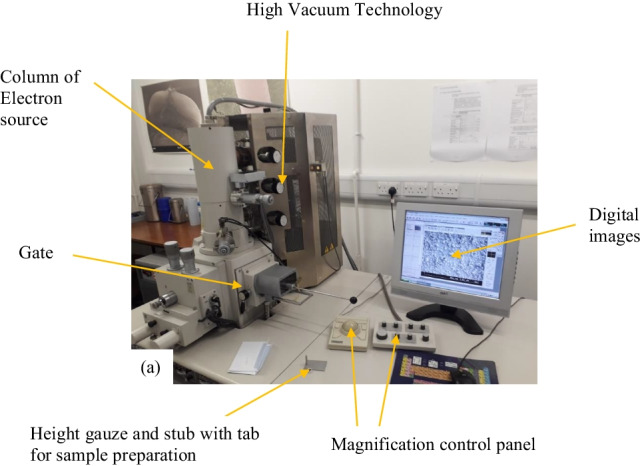
Surface morphology examination using Hitachi S-4300 FE scanning electron microscope

Hitachi S-4300 FE scanning electron microscope (SEM) was utilised for determining the surface morphology of the plasma-treated surface of the wool and cotton fabric samples. Figure 6 depicts the complete SEM system. For analysis, the sample was attached to a rod with a knob that further carries the sample stage to the source of electrons called the rapid sample exchange chamber. The high voltage (HV) mode is set ON and the electrons are generated in the test chamber that bombards the test samples and creates the image on the coordinated digital screen. The image resolution, focus, brightness, darkness and magnification are adjusted with the help of the control panel as shown in Fig. 6. The image is worked upon in TV mode, and a high-quality image is captured on an appropriately generated image. Thereafter, the HV mode is switched off, the magnification is lowered to a minimum of 50 k, and the sample is returned to the home stage in the chamber by external controls. As the air pressure is lowered, the gate opens and the sample slides out with the help of a rod with the knob.

### Fastness tests of inkjet-printed fabrics

The fastness properties against wash, light and rubbing were tested on the wool and the cotton fabric samples inkjet printed with herbal inks, based on the BS ISO 105 C06-A2S, BSI ISO 105 B02 and BS EN ISO 105-X12:2016 respectively (The British Standards Institution 2010) (The British Standards Institution 2014) (The British Standards Institution 2016).

## Results and discussion

The effect of the plasma surface modification on wool and cotton fabrics was examined using ATR-FTIR, SEM, drop-test and surface contact angle analysis as elaborated herein.

### Wettability of wool and cotton fabrics

A surface contact angle being less than 90° the surface is termed hydrophilic, and a surface contact angle being more than 90° the surface is termed hydrophobic. As demonstrated in Table 4, the surface contact angle of wool fabric treated with air plasma has remarkably dropped from 145.84° to 41.35°. Likewise, the surface contact angle of PST cotton fabric has dropped from 146.35° to 80.43°. Hence, it could be assertively established that plasma surface modification has enhanced the wettability of the wool and cotton fabrics, facilitating inkjet printing with herbal inks. Moreover, the coefficient variance (CV) denotes low variation as depicted in Table 4, implying a dependable estimate. The data given in Table 4 is of wool and cotton fabric plasma surface treated for 3 min with 100 W plasma power.

**Table 4:** The surface-contact angle (CA°) mean value as obtained on the optical tensiometer

Fabric name	Contact angle (CA°) mean value	Standard deviation	CV
Untreated wool	145.84	10.43	0.07
Treated wool	41.35	9.76	0.19
Untreated cotton	146.35	13.10	0.09
Treated cotton	80.43	10.21	0.14

### Drop-test appraisal

**Fig. 7:**
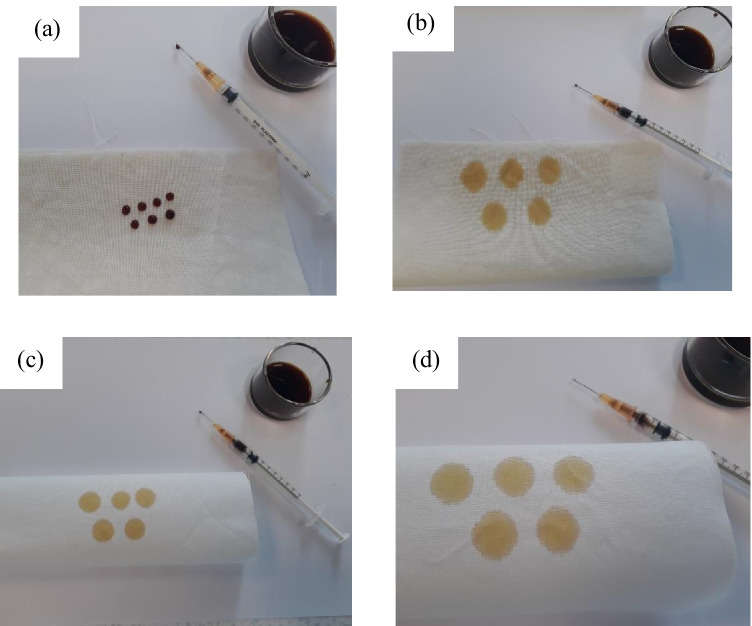
Drop-test on **a** untreated wool and **b** plasma treated wool, **c** untreated cotton and **d** plasma treated cotton

In addition to the contact angle measurement, a drop-test was performed with the water-based herbal ink constituted from the flame of the forest herb as an exemplar. The droplets obtained on the untreated wool and cotton fabrics were observed for comparative purposes. The PST-treated wool and cotton fabrics readily absorbed the herbal ink forming a large area of dispersion than untreated wool and cotton fabrics as demonstrated in Fig. 7a, b, c, and d.

### ATR-FTIR spectral analysis

**Fig. 8:**
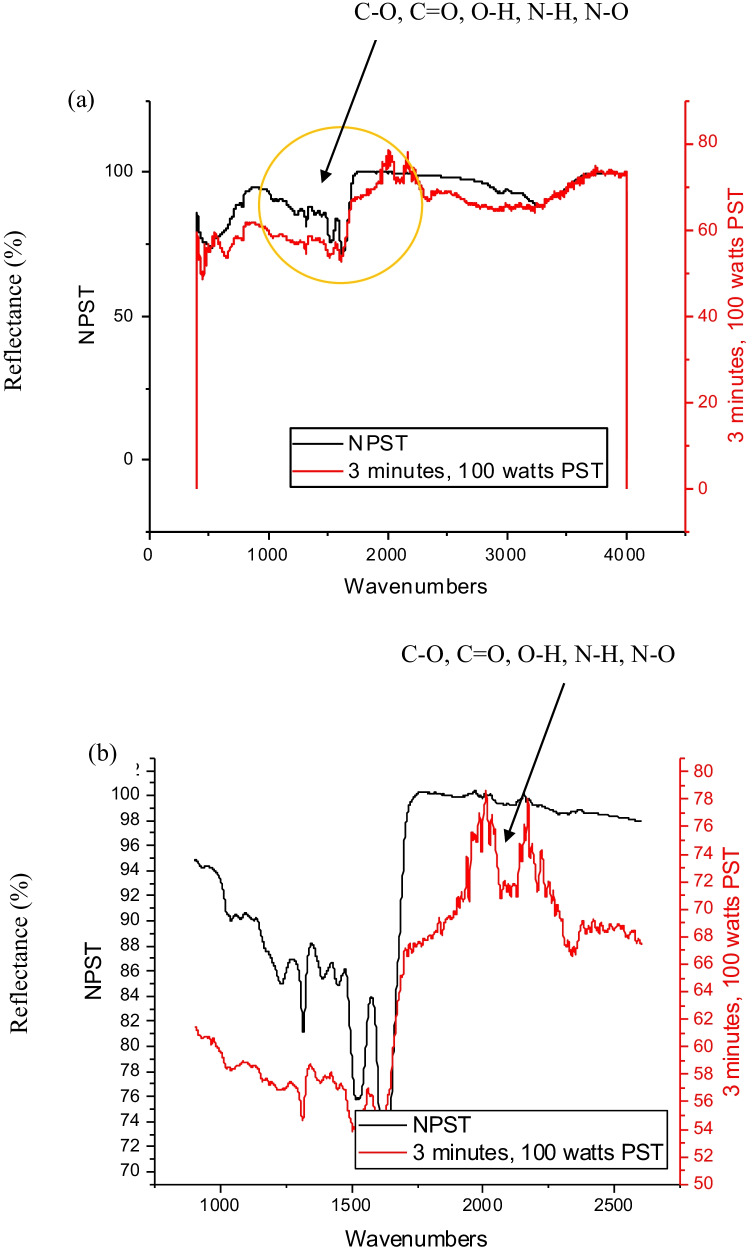
ATR-FTIR analysis, **a** air plasma-treated and untreated plain weave wool fabrics, and **b** extended view

**Fig. 9:**
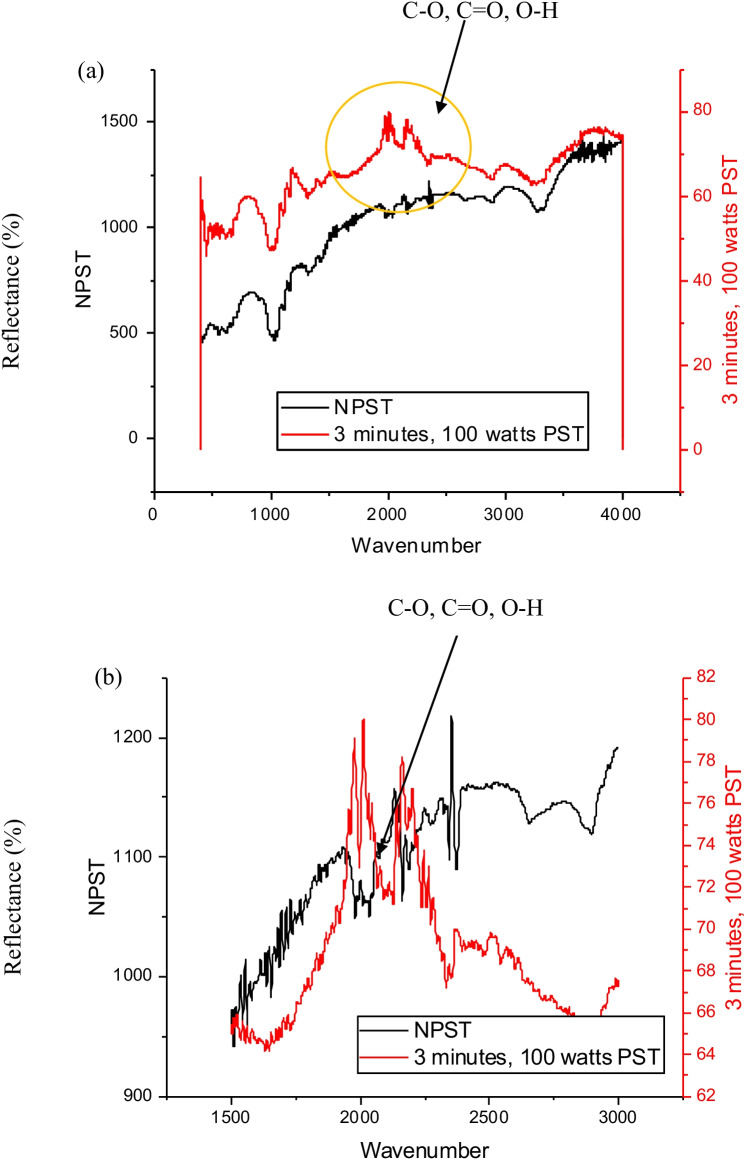
ATR-FTIR analysis, **a** air plasma-treated and untreated plain weave wool fabrics, and **b** extended view

The air plasma surface modification of wool and cotton fabrics was performed at 100 W for 3 min. The results obtained were graphed on OriginLab 2018b as illustrated in Figs. 8a and b and 9a and b for plasma-treated and untreated wool and cotton fabrics, respectively.

The 3-min plasma-treated wool fabric exhibited a strong peak of C–O stretch at the wavenumber of 1313.13 cm^−1^, a strong peak of N–O asymmetric stretch at 1514.91 cm^−1^ and a moderate peak of N–H bend 1° amine at 1608.19 cm^−1^ of wavenumber. The characteristic peaks observed on wool fabrics enable strong hydrogen bond formation at the plasma-induced functional groups namely C–O stretch as demonstrated in Fig. 8a and b, with a yellow circle. Overall, for the wool fabric wavenumbers from 1000 to 2500 cm^−1^ are indicating a significant and major shift of functional groups that correspond to free hydroxyl, carbonyls and H-bonded phenols and amines. The functional groups are additional anchoring sites available for bond formation with chromophores on subsequent inkjet printing. The elevated peak groups are contributed by air plasma surface modification. It implies higher colour absorbance and fastness properties of the resultant herbal fabrics.

The plasma-treated cotton fabric showed a strong peak of = C–H bend at the wavenumber of 982.94 cm^−1^ and a strong peak of C–O stretch at 1102.46 and 1156.92 cm^−1^ wavenumbers. Also, a moderate peak of C–H stretch (in-ring) at 1425.63 cm^−1^ of wavenumber and a strong peak at 3270.57 cm^−1^ wavenumbers of O–H, H-bonded were observed. The characteristics peaks observed on cotton fabrics enable strong hydrogen bond formation at the plasma-induced functional groups namely C–O and O–H stretch as demonstrated in Fig. 9a and b, with a yellow circle. In summary, the cotton fabric wavenumber band from 1500 cm^−1^ to 3000 cm^−1^ implies a substantial and main shift of functional units that correspond to free hydroxyl and carbonyls. Naturally, the functional groups are additional anchoring sites available for bond formation with chromophores on subsequent inkjet printing. The elevated peak groups are owing to air plasma treatment.

### Assessment of surface morphology

**Fig. 10:**
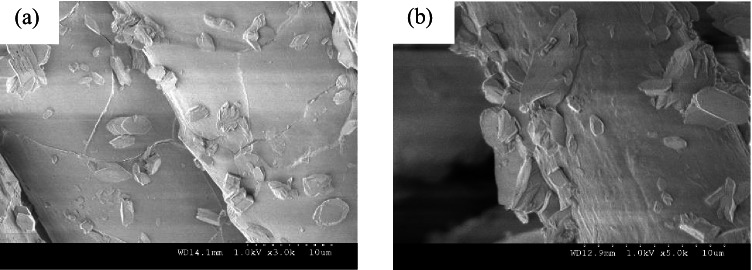
SEM images of wool fabrics, **a** untreated, and **b** 3 min, 100 W PST wool fabrics

**Fig. 11:**
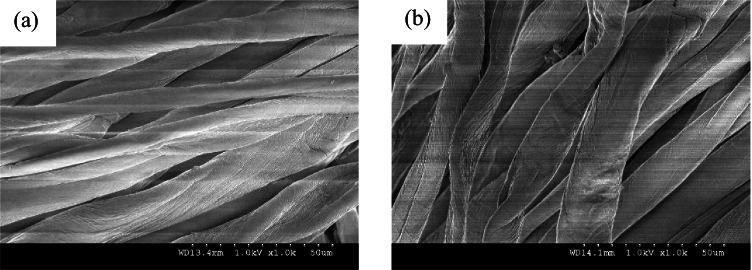
SEM images of cotton fabrics, **a** untreated, and **b** 3 min, 100 W PST cotton fabrics

The PST wool and cotton fabrics were examined on SEM for change in surface morphology as illustrated in Figs. 10a and b and 11a and b correspondingly.

The air plasma surface modification of the wool fabric has further loosened and peeled the scales on the wool fibre structure, as per SEM images demonstrated in Fig. 10a and b for untreated and 3-min PST wool fabric at 100 W of plasma power, respectively, thereby preparing the fabric for enhanced absorption during further pre-treatment process. It would sequentially propel clarity of digitally printed final fabrics, absorption values (K/S) and fastness properties. Turning now to the study of PST cotton fabrics as noted on SEM.

The untreated and PST cotton fabrics treated at 100 W for 3 min are displayed in Fig. 11a and b, respectively. The SEM images of the cotton fabrics after surface modification with plasma appear shrunk and clean thereby enhancing its characteristic convolutions and crease appearance.

**Fig. 12:**
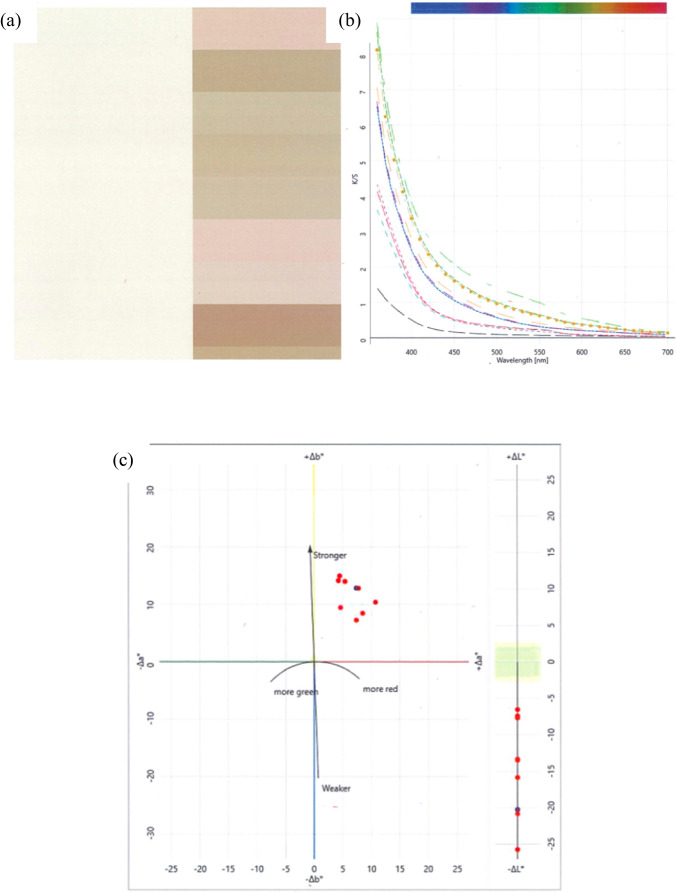
Graphs of study with Group A wool fabrics, **a** colour patch, **b** colour values and **c** colour difference

**Fig. 13:**
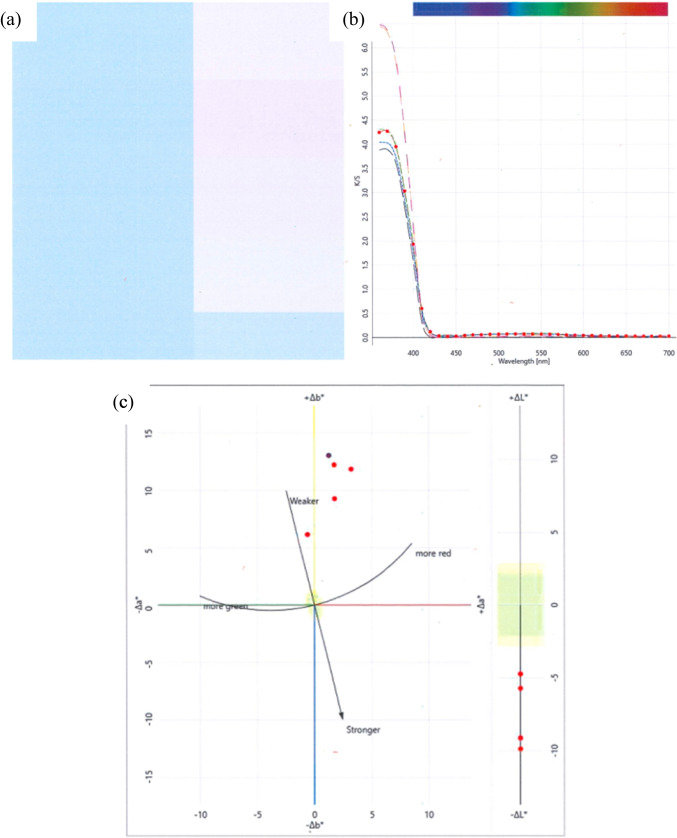
Graphs of study with group B cotton fabrics, **a** colour patch, **b** colour values and **c** colour difference

The deductions accumulated from the above section on the evaluation of plasma-treated fabrics indicate that plasma surface treatment was effective on both wool and cotton fabrics. After air plasma treatment, the treated wool and cotton fabrics were biomordanted with black cherry stem (BCS) solution and pre-treated with guar gum (GG) slurry together referred to as herbal pre-treatment (HPT). The colour values obtained are illustrated in Tables 5 and 6 for groups A and B referring to wool and cotton fabrics individually. The illustrations of the colour patches, colour values and colour difference graphs with Group A and B fabrics are given in Figs. 12a, b, c and 13a, b, c for wool and cotton fabrics, respectively.

**Table 5:** Colour values obtained on group A wool fabrics

FN	PT	HC	L*	a*	b*	K/S	ΔΕ
OWF	–	–	86.84	− 0.39	11.03	0.5020	–
A1, PST and HPT	HPT	M	78.64	8.11	19.49	1.5394	14.26
A2	CPT	M	65.49	7.38	28.83	3.5175	25.67
A3	CPT	K	72.98	3.88	25.15	2.4864	19.90
A4	CPT	Y	70.48	5.01	25.02	2.8142	21.83
A5	CPT	Y	72.85	4.09	25.96	2.5585	20.61
A6, PST and HPT	HPT	M	78.91	7.00	18.26	1.3999	12.73
A7, PST and HPT	HPT	M	79.79	4.25	20.48	1.6283	12.40
A8	CPT	M	60.58	10.31	21.42	3.8543	29.76
A9	CPT	Y	66.06	6.99	23.87	3.3563	25.11

**Table 6:** Colour values obtained on Group B cotton fabrics

FN	PT	HC	L*	a*	b*	K/S	ΔΕ
OCF	–	–	96.64	3.84	− 15.49	1.5747	–
B1, PST and HPT	HPT	M	87.54	5.10	− 2.45	1.9337	15.95
B2, PST and HPT	HPT	M	86.78	7.04	− 3.64	1.9235	15.75
B3, PST and HPT	HPT	M	87.52	5.57	− 3.27	1.7863	15.35
B4	CPT	Y	90.92	5.60	− 6.23	2.3736	11.03
B5	CPT	M	91.91	3.24	− 9.34	2.3151	7.78

### Colour values of inkjet-printed fabrics

Table 5 and Fig. 12a, b and c indicate that the maximum Δ*E* of 14.26 was noted with *M* colour herbal ink on PST and HPT wool fabric and 29.76 Δ*E* on commercially pre-treated wool fabric. Still, the present study raises the possibility that herbal inks could be formulated with herbs and successfully applied on prepared wool fabrics acquiring the tabulated values to prevent the perils arising from CPT to the environment and human health alike.

Table 6 and Fig. 13a, b and c indicate that the maximum Δ*E* of 15.95 was noted with *M* colour herbal ink on PST and HPT cotton fabric whereas the Δ*Ε* of 11.03 was obtained on CPT fabric. These findings have important implications for developing herbal inks and utilising them for digitally printing the prepared cotton fabrics.

### Fastness properties of the inkjet-printed fabrics

What is interesting about the data in Table 7 is that both the wash fastness ratings to multifibre fabric staining and colour change are very good for the wool fabric inkjet printed with herbal ink. The observed increase in wash fastness test ratings could be attributed to the applied plasma surface modification. Plasma surface modification stimulates C–C and C–H bonds to dissipate. These carbon radicals react with oxygen atoms and radicals in plasma or hydrogen in the air forming C–OH, C = O and C–O functional groups (Chi-Wai and Wai-Shan 2018), thereby providing extra affixing sites for the chromophores to form a large complex within the fabrics structures that is fast to wash. Hence, the improved ratings are observed.

**Table 7:** Wash fastness test result of group A wool fabrics

FN	HC	Multi-fibre test fabric staining						CC
		DICEL	BUMC	Ny	Py	Ay	WW	
A1	K	5	5	5	5	5	5	4–5
A2	Y	5	5	5	5	5	5	5
A3	M	5	5	5	5	5	5	4–5
A4	M	5	5	5	5	5	5	4–5

DICEL is secondary cellulose acetate and BUMC is bleached un-mercerized cotton. Ny is Nylon 6.6 and Py is polyester (Terylene). Ay is acrylic (Courtelle) and WW is wool worsted. CC implies colour change

Data from Table 8 shows that both the wash fastness ratings to multifibre fabric staining and colour change are improved. The wash fastness to colour change is enhanced from poor (1–2 for untreated) to moderate (2–3, 3 and 3–4) for the cotton fabric inkjet printed with herbal ink. The observed increase in wash fastness test ratings could be attributed to the utilized plasma surface modification.

**Table 8:** Wash fastness test result of Group B cotton fabrics

FN	HC	Multi-fibre test fabric staining						CC
		DICEL	BUMC	Ny	Py	Ay	WW	
B1	M	5	5	5	5	5	5	2–3
B2	M	5	5	5	5	5	5	3
B3	Y	5	5	5	5	5	5	3
B4	M	5	5	5	5	5	5	3–4

The lightfastness test results were examined with the blue wool standard; the appraisal is as follows.

The data points in Table 9 state that the A3 and A4 have gained acceptable lightfastness probably due to PST.

**Table 9:** Lightfastness test result for Group A set of wool fabrics

FN	HC	Ratings
A1	K	2
A2	Y	1
A3	M	4
A4	M	5

The records from Table 10 denote that the group B cotton fabrics were anticipated to have good to excellent lightfastness values owing to PST; however, it is observed to be steadfastly fair. Together with information in Tables 9 and 10, the same trends are observed in lightfastness ratings acquired for wool and cotton fabrics, respectively. Also, it is of common knowledge in the field of colouration with plant extracts that shade drying is advocated for the finished garments so that possible fading on exposure to sunlight is evaded.

**Table 10:** Lightfastness test result for Group B set of cotton fabrics

FN	HC	Ratings
B1	M	3
B2	M	3
B3	Y	3
B4	M	1

What is interesting about the data shown in Table 11 is that both wet and dry rub fastness ratings are improved to excellent; the change is credited to plasma surface modification of the wool fabric.

**Table 11:** Group A wool fabrics rub fastness

FN	HC	Rub fastness	
		Wet	Dry
A1	M	5	5
A2	M	4	5
A3	K	4–5	5
A4	Y	4–5	5
A5	Y	4–5	5
A6	M	5	5
A7	M	5	5
A8	M	4	5
A9	Y	4–5	4–5

Similarly, it is apparent from Table 12 that both the wet and dry rub fastness rating is enhanced from being very good to excellent evidently due to plasma surface modification of cotton fabric. Conclusively, the results of this investigation show that the wool and cotton fabrics inkjet printed with herbal inks have good wet and dry rub fastness to staining, and it could be improved to excellent with plasma surface modification of the wool and cotton fabrics. This is for the reason that plasma surface modification enhances the anchoring sites of colourant to the fabric rendering it fast to abrasion. The additional polar functional groups such as C = O, C–O and OOH are available for adhering to the chromophore forming strong hydrogen bonds that improve the fastness properties of the treated fabrics.

**Table 12:** Group B cotton fabrics rub fastness

FN	HC	Rub fastness	
		Wet	Dry
B1	M	4–5	5
B2	M	5	5
B3	M	5	5
B4	Y	5	5
B5	M	5	5

### Quality assessment of inkjet-printed images

The wool and cotton fabric samples inkjet printed with plant-based inks were evaluated for the quality of print with regard to ruggedness and sharpness of lines and edges. The solid pattern printed surface was examined for its evenness of print and colour. The representative Quebracho red bark extract colour (M) ink is selected for analysing the print quality of plasma-treated in comparison to the untreated wool and cotton fabric samples individually.

**Fig. 14:**
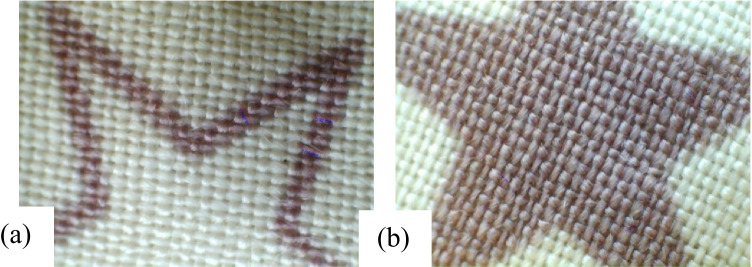
Print quality as obtained on NPST wool fabric inkjet printed with Quebracho red ink. **a** Printed line and **b** solid pattern

**Fig. 15:**
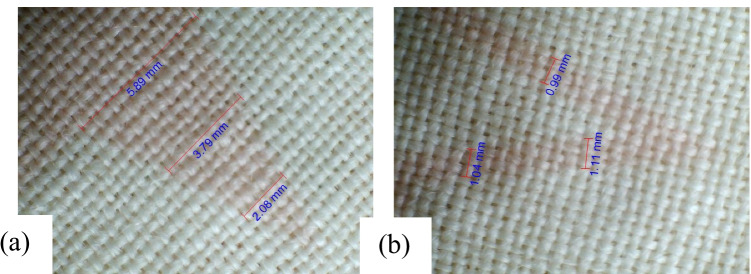
Print quality as obtained on PST wool fabric inkjet printed with Quebracho red ink. **a** Solid pattern and **b** printed line

The untreated wool fabric digitally printed with PI1 from the quebracho plant is presented in Fig. 14a and b. The observed uneven line and solid pattern were attributed to patchy pre-treatment. The plasma-treated wool fabric digitally printed with PI3 from the quebracho plant is shown in Fig. 15a and b. Remarkably, uniform printed lines and solid surfaces are seen credited to plasma surface modification technology. The relevant measurements in millimetres (mm) are organised in Table 13.

**Table 13:** Dimensions in millimetres of inkjet-printed line and solid surface on wool fabric as acquired with USB digital microscope

Fabric samples	IPL	IPSP
M, NPST	0.92	3.67
	1.04	3.76
	1.16	4.84
M, PST	0.99	2.08
	1.04	3.79
	1.11	5.89

The IPL is inkjet-printed line, and IPSP is inkjet-printed solid pattern

**Fig. 16:**
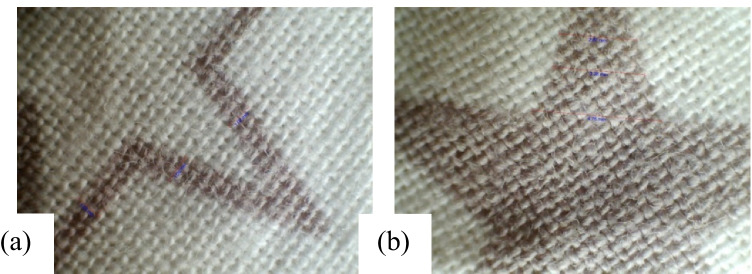
Print quality as obtained on NPST cotton fabric inkjet printed with Quebracho red ink. **a** Printed line and **b** solid pattern

**Fig. 17:**
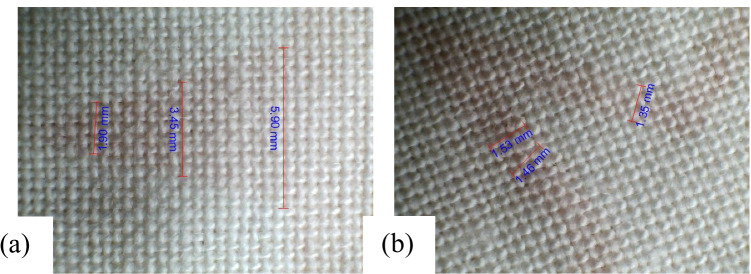
Print quality as obtained on PST cotton fabric inkjet printed with Quebracho red ink. **a** Solid pattern and **b** printed line

In the same manner, the NPST cotton fabric inkjet printed with PI1 from the quebracho plant is offered in Fig. 16a and b. The ragged line and smudged solid pattern were observed owing to sporadic pre-treatment. The PST cotton fabric digitally printed with PI3 from the quebracho plant is shown in Fig. 17a and b. Noticeably, even printed lines and solid patterns are attributed to plasma surface modification and uniformally imparted polar groups on the treated fabric surface. The applicable measurements of millimetres (mm) are shown in Table 14. The print quality assessment was successful as it was able to identify the defective print and the likely reason for it. In summary, these results show that patchy plant-based pre-treatment may be readily prevented to acquire even prints. There was a significant printed quality difference between the plasma-treated and untreated wool and cotton fabric samples. It is distinctly perceptible that the PSM conferred uniformity to resultant prints.

**Table 14:** Dimensions in millimetres of inkjet-printed line and solid surface on cotton fabric as acquired with USB digital microscope

Fabric samples	IPL	IPSP
M, NPST	1.02	2.87
	1.03	3.38
	1.09	4.76
M, PST	1.35	1.90
	1.46	3.45
	1.53	5.90

### Statistical analysis

#### Plasma surface treatment variance and K/S response

The data utilised for ANOVA one-way test analysis for determination of plasma surface treatment variance on K/S response is presented in Table 15.

**Table 15:** Data utilised for analysis of plasma surface treatment variance and K/S response

Fabric details	K/S
PST and HPT	1.3999
	1.6283
	1.5394
	1.9337
	1.9235
	1.7863
CPT	3.5175
	2.4864
	2.8142
	2.5585
	3.8543
	3.3563
	2.3736
	2.3151

**Fig. 18:**
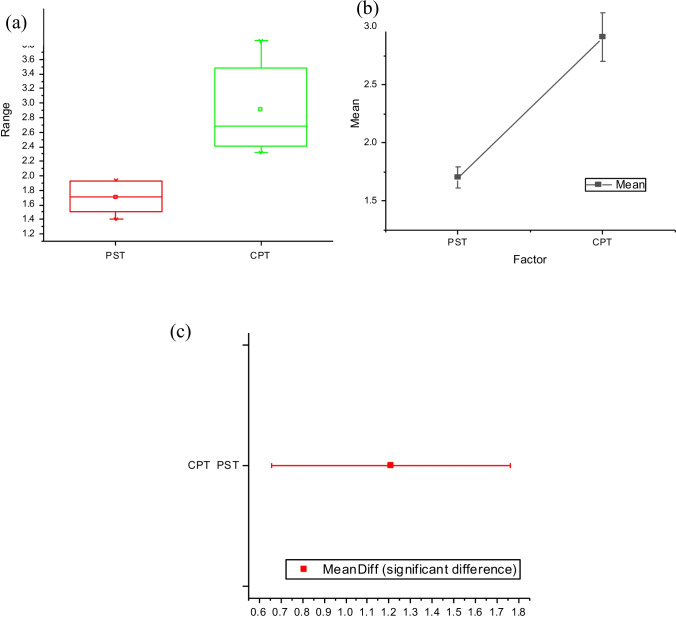
Illustrations of ANOVA one-way test result for plasma surface modification parameter, **a** box plot, **b** standard error of the mean plot, and **c** Bonferroni graph

The results of the statistical assessment are summarised in Table 16 and illustrated in Fig. 18a, b, and c. A positive correlation was found between plasma surface treatments with herbal pre-treatment on the acquired K/S values. This statement is supported by the *p*-value of 0.0004630 which is far less than 0.05 of the significant level. Equally, Bonferroni test demonstrated the Sig = 1 denoting a significant mean difference at the 0.05 level. Moreover, the actual power of 0.99 indicates the data results to be 99% factual.

**Table 16:** Statics gained for plasma surface treatment variance and K/S response

Parameter	Mean	SD	SE	Probability	Sig	Actual power
PST	1.70	0.21	0.08	4.630 E − 4	1	0.99
CPT	2.90	0.58	0.20	4.630 E − 4	1	0.99

SD is the standard deviation of the mean and SE is the standard error of the mean

## Conclusions

In summary, the plasma surface modification of the wool and cotton fabrics was performed ecologically generating no emission and effluents. The following inferences were drawn:

1. The surface contact angle of wool fabric treated with air plasma has remarkably dropped from 145.84° to 41.35°. Likewise, the surface contact angle of PST cotton fabric has dropped from 146.35° to 80.43°. Thereby enhancing the hydrophilicity of the treated fabrics.
2. For the wool fabric, wavenumbers from 1000 to 2500 cm^−1^ and for the cotton fabric wavenumber band from 1500 to 3000 cm^−1^ imply a substantial and main shift of functional units that correspond to free hydroxyl and carbonyls, hence confirming the increase in the polar functional groups due to plasma surface modification.
3. The surface morphology of plasma-treated wool and cotton fabrics appeared more scally and grooved, respectively. This change in structure indicates greater absorbency.
4. The wool and cotton fabrics inkjet printed with herbal inks acquired the maximum colour difference values of 14.26 and 15.95 for red colour herbal ink on wool and cotton fabrics, respectively.
5. The wash fastness to staining and rub fastness was excellent for all the wool and cotton fabrics. The wash fastness to colour change was enhanced to be very good for wool and moderate for cotton fabrics. These results are credited to plasma surface modification. However, the light-fastness was not steadfast; it ranged from poor up to good for plasma surface–treated wool and cotton fabrics inkjet printed with herbal inks. These could be attributed to the uneven herbal pre-treatment which could be overcome. Therefore, shade drying is recommended.
6. The study was validated statistically with an ANOVA one-way test denoting a standard error of 0.08 gained with plasma surface–treated fabrics and a standard error of 0.20 acquired with commercially pre-treated fabrics. The probability values of 4.630 E − 4 and actual power of 0.99 indicate statistically significant results.

**Fig. 19:**
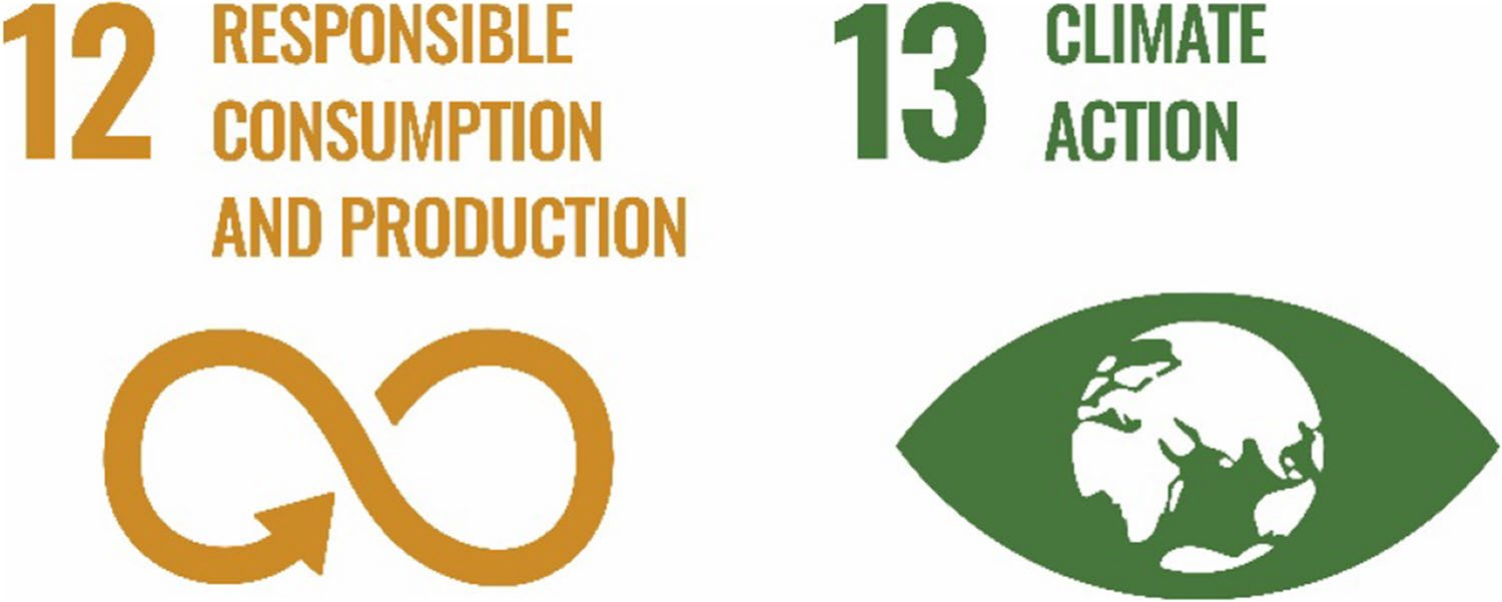
The SDGs fulfilled by research

Overall, it is justified that the plasma surface modification is more advantageous in obtaining the improved colour values and fastness properties. Therefore, the commercial pre-treatment which involves emissions and effluents could be overlooked. Doing so would benefit both environment and human health alike. The research adheres to Sustainable Development Goals as illustrated in Fig. 19 specifically responding to climate action and responsible production and consumption by implementing natural biomaterials from plants and green technologies such as plasma treatment and waterless inkjet printing that create virtually no emissions and effluents hence sustainable. This way, the study contributes toward carbon neutrality.

### Future work

To end, the research paper investigated in-depth the digital printing of wool and cotton fabrics applying plasma surface modification technology and herbal inks, in this manner achieving sustainable solutions to refute emission and effluents from the textile wet processing units implementing synthetic materials. The achieved results were discussed and deducted to be promising. Therefore, in the future scope of research and development, life cycle analysis and examination of functional properties of the resultant herbal fabrics are recommended.

## Data Availability

The authors have duly acknowledged data and materials in the research.
